# Prevalence and risk factors of untreated thyroid dysfunctions in the older Caucasian adults: Results of PolSenior 2 survey

**DOI:** 10.1371/journal.pone.0272045

**Published:** 2022-08-22

**Authors:** Piotr Kocełak, Małgorzata Mossakowska, Monika Puzianowska-Kuźnicka, Krzysztof Sworczak, Adam Wyszomirski, Gabriela Handzlik, Adrian Stefański, Tomasz Zdrojewski, Jerzy Chudek

**Affiliations:** 1 Medical Faculty in Katowice, Department of Pathophysiology, Pathophysiology Unit, The Medical University of Silesia, Katowice, Poland; 2 International Institute of Molecular and Cell Biology, Warsaw, Poland; 3 Department of Geriatrics and Gerontology, Medical Centre of Postgraduate Education, Warsaw, Poland; 4 Department of Human Epigenetics, Mossakowski Medical Research Institute, Polish Academy of Sciences, Warsaw, Poland; 5 Department of Endocrinology and Internal Diseases, Medical University of Gdańsk, Gdańsk, Poland; 6 Faculty of Medicine, Department of Adult Neurology, Medical University of Gdańsk, Gdańsk, Poland; 7 Medical Faculty in Katowice, Department of Internal Medicine and Oncological Chemotherapy, Medical University of Silesia, Katowice, Poland; 8 Division of Preventive Medicine and Education, Medical University of Gdańsk, Gdańsk, Poland; Phramongkutklao College of Medicine, THAILAND

## Abstract

**Introduction:**

To determine the prevalence of treated and untreated thyroid dysfunction and to identify factors associated with increased risk of undiagnosed thyroid dysfunction in older adults.

**Methods:**

The population of 5987 community-dwelling Polish Caucasian seniors aged 60 years and above who participated in the PolSenior 2 study (2018–2019). Population-based cross-sectional multidisciplinary study in design. Data from structured questionnaires, geriatric tests, and scales were obtained from all study participants who underwent anthropometric and blood pressure measurements during three home visits. Assessment of thyroid function was based on TSH serum measurements.

**Results:**

The prevalence of thyroid dysfunction in the Polish population aged 60 years or above was estimated at 15.5% (21.5% in women and 7.2% in men), with 3.2% of undiagnosed individuals among them. The prevalence of hypothyroidism and hyperthyroidism in the studied group was 13.9% (19.4% in women and 6.3% in men) and 1.6% (2.1% in women and 0.9% in men) respectively, untreated hypothyroidism was revealed in 21.9% (in 160 out of 732 subjects) and untreated hyperthyroidism in 34.2% of subjects (in 41 out of 120 participants). In multiple regression analysis independent risk factors for thyroid disorders being untreated were older age (> 75 years), male sex, a low education level (primary or lower), and low utilization of medical services.

**Conclusions:**

One-fifth of Polish Caucasian seniors with hypothyroidism and one-third with hyperthyroidism are untreated. Older, poorly educated and rarely utilizing medical services seniors, especially men, are more frequently untreated for thyroid dysfunction and some of them do not benefit from contemporary achievements in medicine.

## Introduction

Thyroid dysfunction, mostly hypothyroidism, is the leading endocrinopathy in older adults [1], frequently with clinical presentation diverse from that in younger ones [2]. Current clinical guidelines recommend the same cut-off points for thyroid hormones in the diagnosis of thyroid dysfunction, irrespective of age, sex, race, and population iodine supply [3,4], which may explain vast differences in the prevalence of thyroid dysfunction reported in different populations [5]. One of the major points is the regional variability of iodine supply resulting in the differences in populational TSH distribution. Low iodine intake is associated with a shift of TSH towards lower values, and an increase in the prevalence of hyperthyroidism [6], while the higher intake is related to population iodine supply, with a shift towards higher TSH values [7]. Nowadays, hypothyroidism associated with severe iodine deficiency is rarely seen in Europe. The policy of iodine fortification with the adoption of iodine-fortified salt, which currently covers about 71% of the world’s population [8] may force a change in reference ranges of TSH [9].

The prevalence of thyroid dysfunction was analyzed in the general European population in the years 1975–2012. Meta-analysis of 7 population-based and cohort studies [10] covering Western European countries (excluding individuals with already diagnosed thyroid diseases) revealed that the overall prevalence of thyroid dysfunction was estimated at 6.7% (95%CI: 6.5–6.9), including hypothyroidism– 4.9% (95%CI: 4.8–5.1) and hyperthyroidism– 1.7% (1.7–1.9). Of those 80.1% of disturbances were subclinical. No sub-analysis for older adults was done. In summarized observational studies, the incidence rate of thyroid dysfunction was estimated at 420 / 100,000 / year in women and 85 / 100,000 / year in men [10].

Thyroid dysfunction is highly prevalent in older adults. Based on the PolSenior1 study, performed between the years 2007–2011 the frequency of thyroid dysfunction in the Polish population aged 55 yrs. or over was almost 11%, including hypothyroidism in 8%, and hyperthyroidism in 3% of study subjects [11].

Notwithstanding, much higher numbers were recently shown in a cohort of 5,392 Americans aged 65 yrs. or over, participants of the Atherosclerosis Risk in Communities (ARIC) study performed in the years 2011–2013 [12]. Thyroid dysfunction was diagnosed in 24.9% of the study population, including 16.9% and 0.1% already treated for hypothyroidism and hyperthyroidism, respectively. Among untreated subjects, subclinical hypothyroidism (6.1%) was about seven times more prevalent than overt hypothyroidism (0.8%), while subclinical hyperthyroidism (0.8%) was about three times than overt hyperthyroidism (0.3%).

The data from Central and Eastern Europe are sparse. A cross-sectional case-control study carried out in a single tertiary referral centre in Bulgaria [13] included in the meta-analysis of the prevalence of hypothyroidism, suggests a higher prevalence of hypothyroidism in the general population in Eastern than in Western Europe. A similar observation was presented in another meta-analysis [14], which covered the prevalence of only undiagnosed hypothyroidism in the general population in Europe. It has included mostly population-based and cohort studies from old European Union countries published from 2008 to 2017 [14]. The prevalence of undiagnosed hypothyroidism in an older population (≥ 65 yrs.) was greater than in the younger cohort and estimated at 6.6% (95% CI 2.5–12.4), including subclinical 5.1% (95%CI: 3.3–7.3) and overt disease 1.1% (95%CI: 0–3.4). The published analysis of thyroid function in the PolSenior 1 project has omitted this aspect [11] that is important for public health policies and programs, as health consequences related to untreated thyroid disturbances can be serious and, in some cases, life-threatening. The most important are cardiovascular diseases, diabetes, and rarely myxedema coma or thyroid storm [15–17].

The aim of this study was to analyze the frequency of treated and untreated thyroid dysfunction and to identify factors associated with increased risk of undiagnosed thyroid dysfunction in older adults, based on a representative national cohort.

## Materials and methods

The PolSenior 2 study enrolled 5987 subjects aged 60 years and above, a representative of the community-dwelling Polish population. The study was carried out from January 2018 to December 2019 by 507 nurses deliberately trained for study purpose and specificity. The study procedures included structured questionnaires, geriatric tests and scales, as well as anthropometric and blood pressure measurements performed during three home visits. Participants were asked to show all medicines (including products containing levothyroxine or antithyroid drugs) taken during the last two weeks. A multistage stratified and clustered random sampling method was used to recruit subjects. The population aged 60+ was divided into 78 strata. During the first stage, municipalities were drawn in each stratum with probability proportional to the size (PPS) of the population in a particular municipality. In the next step, villages and cities were drawn in the previously selected municipalities. Finally, participants were drawn from the set of villages and cities. Roughly, equal numbers of study participants were drawn in each 5-year age and sex group. Such study design was intended to increase the statistical power of analyses performed in the oldest age groups. The detailed sampling procedure, as well as the construction of the study questioners, have been described previously [18].

Blood samples were donated by 5825 participants (97.3%). Blood samples were not obtained from 162 participants due to refusal or difficulty in venepuncture. Thyroid-stimulating hormone (TSH) was assessed in 5775 (99.1%) serum samples in a single central laboratory (Brus Laboratory in Gdynia) using Atellica Solution Immunoassay & Clinical Chemistry Analyzers, and reagents from Siemens Healthineers. The reference range for TSH was 0.55–4.78 mIU/L. In the remaining 50 cases, TSH was not evaluated due to insufficient serum sample volume.

The analysed group (N = 5800; 93.5% of the PolSenior2 cohort) included all subjects with measured TSH concentration and 25 participants treated with levothyroxine or antithyroid drugs without measured TSH level.

All the subjects included in the study gave written informed consent for participation in the study. The study was approved by the ethics committee of the Medical University of Gdansk. The study was conducted according to the Declaration of Helsinki requirements.

### Data analysis

**Fig 1:**
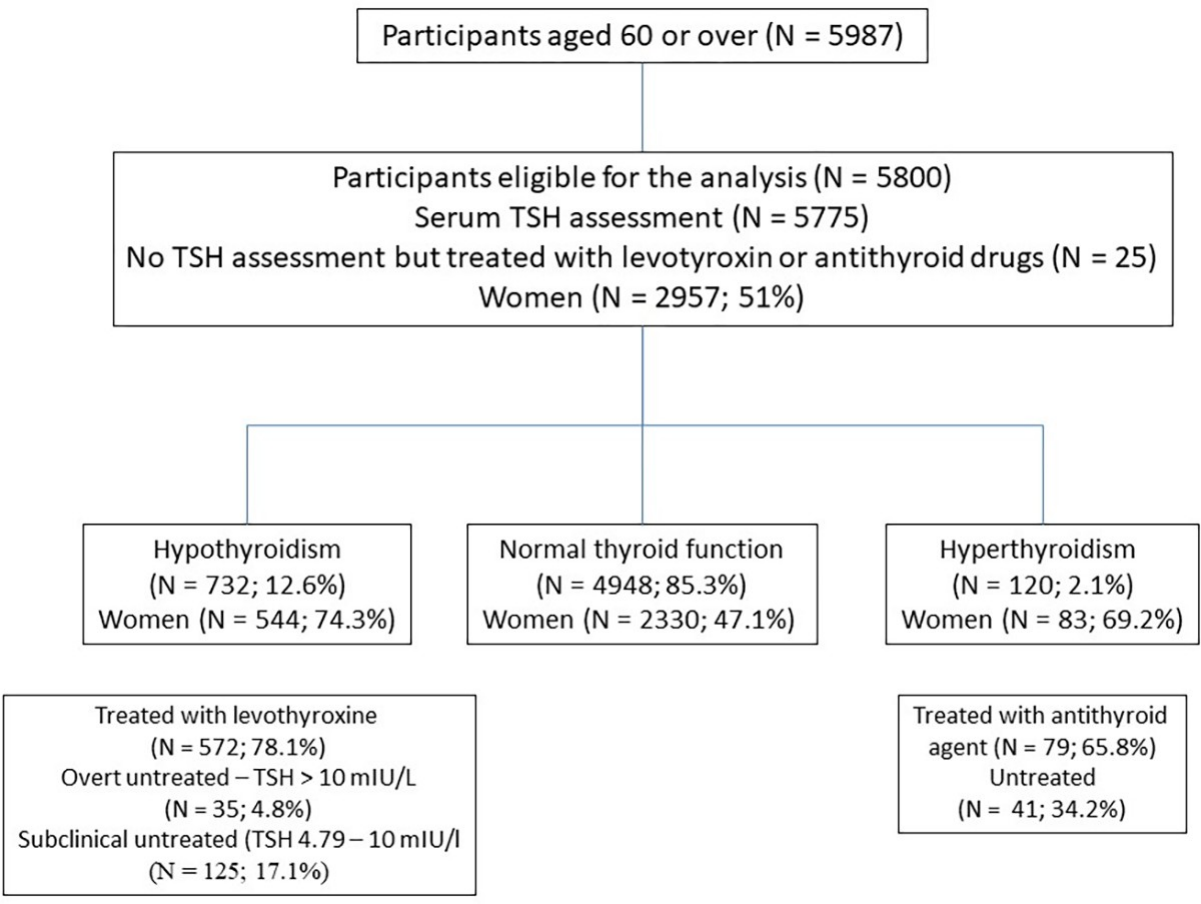
Chart flow of the study.

Thyroid disorders were classified as 1) hypothyroidism if levothyroxine-containing medicine was used or when elevated (> 4.78 mIU/L) TSH level was present in participants not using antithyroid drugs [19], 2) clinically significant hyperthyroidism if the antithyroid drug was taken or decreased (<0.1 mIU/L) TSH level, was found in people not using levothyroxine [20,21]. (Fig 1).

The subgroup of untreated hypothyroidism (not using levothyroxine preparations) was divided into subclinical (TSH ≤ 10 mIU/L) and overt hypothyroidism (TSH > 10 mIU/L). Among subjects treated with levothyroxine preparations and assessed TSH concentration, the percentage of subjects with elevated TSH levels (> 4.78 mIU/L) was calculated.

In the subgroup with clinically significant hyperthyroidism, the percentage of people using and not using antithyroid drugs was calculated, and among those treated with antithyroid drugs, the percentage of subjects with persistent hyperthyroidism despite the treatment (TSH <0.1 mIU/L) was also evaluated.

Treated subjects both with levothyroxine or antithyroid drugs with missing TSH assessments were excluded from the analysis of the treatment efficacy.

The analysis included: sex, place of residence, marital and living status, educational level, years of education, type of work currently or in the past (grouped into ‘blue collar’ or farmer, ‘white collar’, and other), years of work, current professional activity, use of the Internet, frequency of visits to the general practitioner, visits to specialists and hospitalization during last 5-year period, economic status, the Lawton Instrumental Activities of Daily Living (iADL) [22], Mini-Mental State Examination (MMSE), corrected by age and education according to Mungas [23] and Geriatric Depression Scale (GDS).

### Statistical analysis

Statistical analysis and data visualization were conducted using the R statistical package (R Core Team, version 3.6.3.). No imputation of missing data was performed. Sampling weights were included in calculations of proportions (prevalence rates) and 95% confidence intervals to account for the complex survey design. The post-stratification procedure was used to match the age-sex sample distribution to the national population. We applied unweighted logistic regression to assess the relationship between untreated thyroid dysfunctions and a set of risk factors. Potential risk factors were selected based on literature and experts’ knowledge. Statistically significant factors (p-value < 0.05) were included in a multivariable model followed by a step-wise backward selection procedure. Regression coefficients were expressed as odds ratios with a 95% confidence interval. The between-group differences were tested using the unpaired t-test for quantitative variables and the chi-square test in the case of categorical data. The 2-tailed tests were carried out at a significance level of p < 0.05.

## Results

### Distribution of TSH values

**Fig 2:**
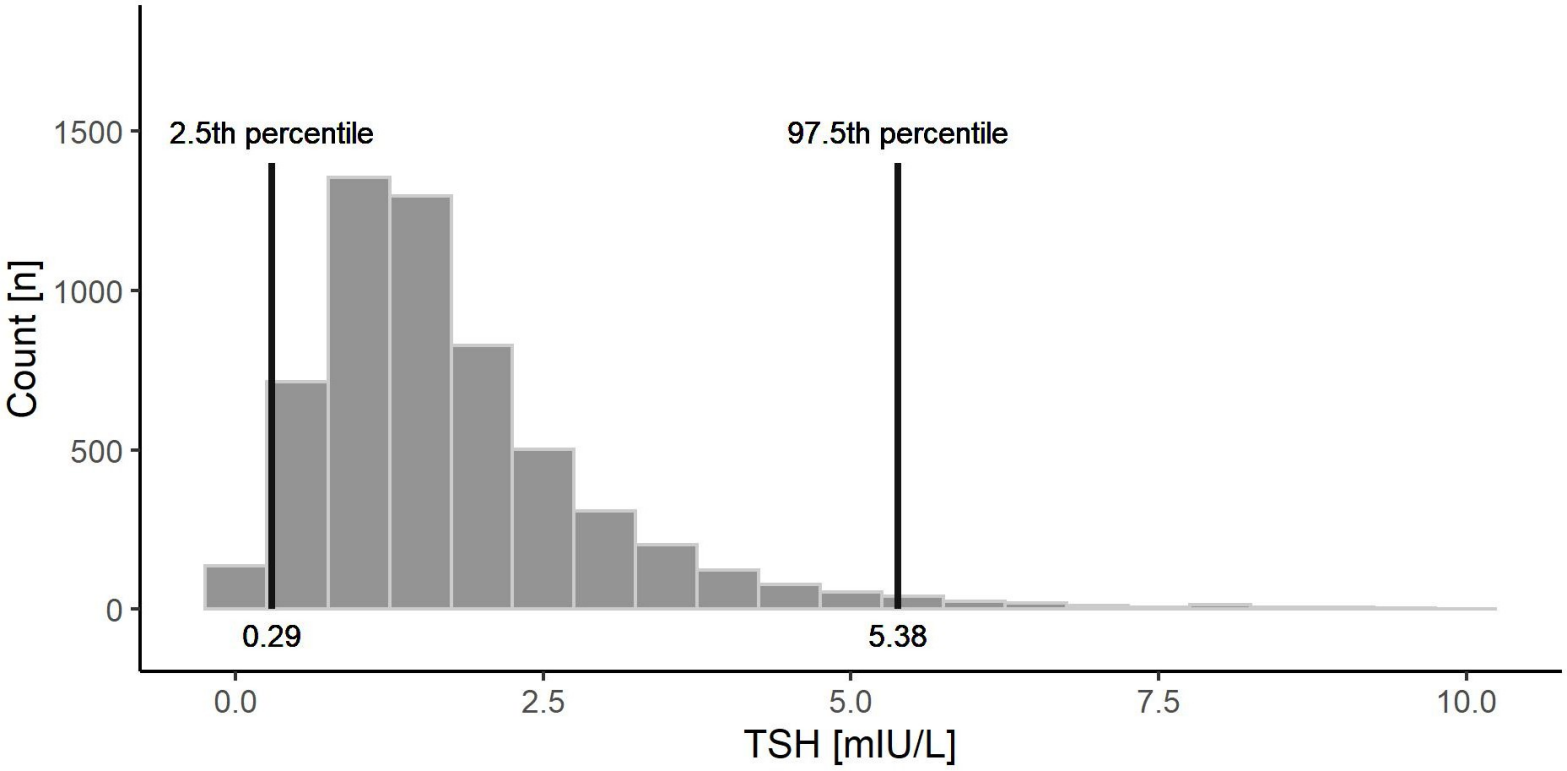
Distribution of serum TSH levels in the study cohort with marked values for 2.5 and 97.5 percentiles.

A histogram of TSH values in our study population is presented in Fig 2. The 2.5–97.5% range was 0.28–5.38 mIU/L. Such an established population-specific reference range was wider than provided by the manufacturer (0.55–4.78 mIU/L).

### The prevalence of thyroid dysfunction

**Fig 3:**
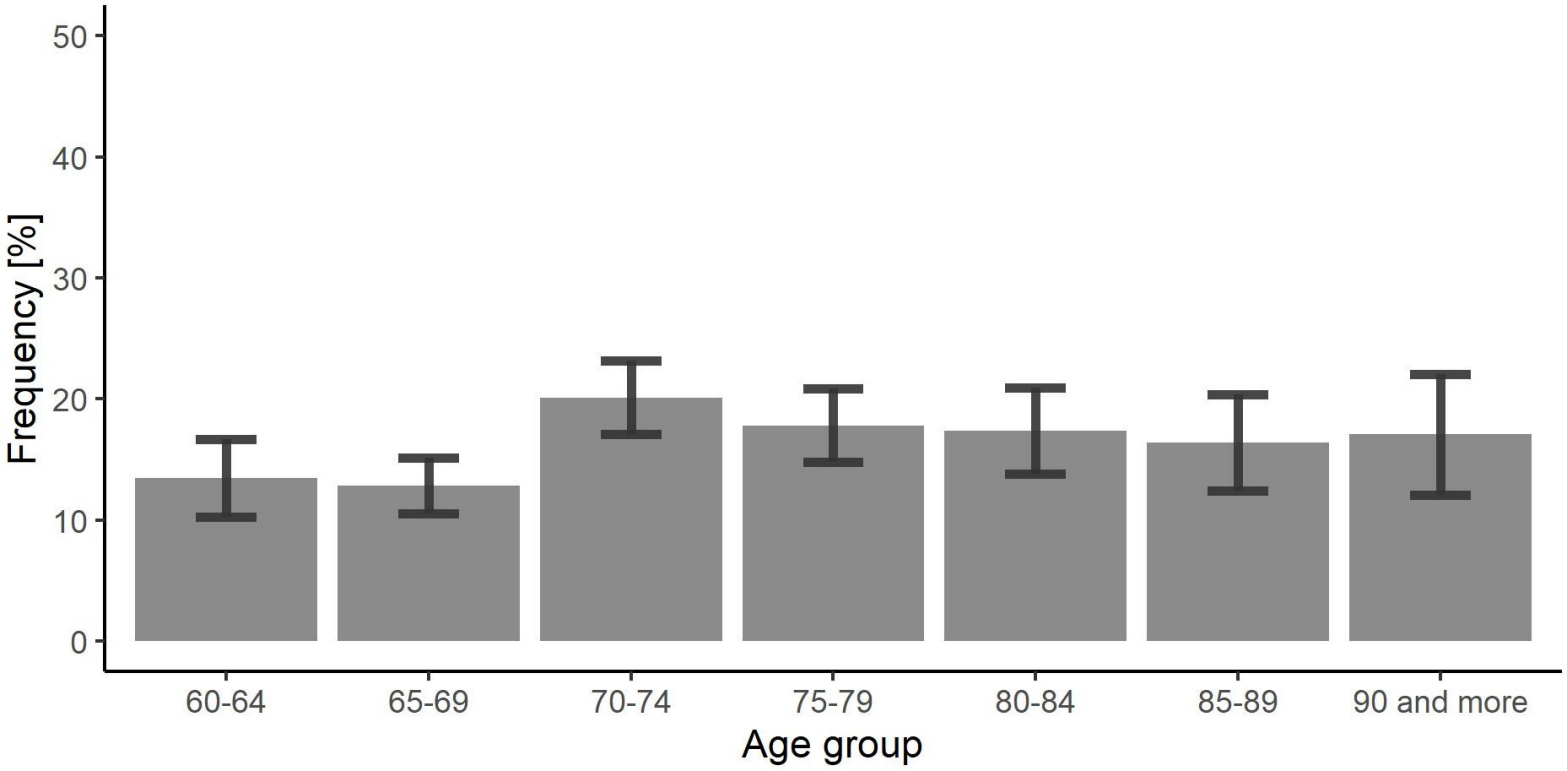
The frequency of thyroid dysfunction across age cohorts.

Treated and untreated thyroid dysfunction was found in 849 participants (625 women and 224 men). Based on this data the prevalence of thyroid dysfunctions was estimated at 15.5% (95%CI: 14.1–16.9) in the whole Polish population aged 60 yrs. or above, 21.5% (95%CI: 19.2–23.8) in women and 7.2% (95%CI: 6.0–8.4) in men (p < 0.001). The frequency of thyroid dysfunction across the analyzed age cohorts was similar (Fig 3).

### Hypothyroidism

Hypothyroidism was found in 732 participants (544 women and 188 men). Based on this data, the prevalence of hypothyroidism was estimated at 13.9% (95%CI: 12.5–15.4) in the whole Polish population aged 60 yrs. or above, 19.4% (95%CI: 17.1–21.7) in women and 6.3% (96%CI: 5.1–7.5) in men (p < 0.001). The disorder was untreated in 21.9% cases (N = 160), including 100 women (18.4%) and 60 men (31.9%), (p < 0.001). The majority (N = 125; 78.1%) of subjects with untreated hypothyroidism presented subclinical dysfunction (TSH ≤ 10 mIU/L), with no difference between women and men (N = 83–85% and N = 42–70%, respectively; p = 0.055). The subgroup with TSH concentration > 10 mIU/L consisted of 35 participants, 17 women and 18 men (4.8%, 3.1% and 9.6% of subjects with untreated hypothyroidism, respectively; p < 0.001).

Among 572 individuals treated with levothyroxine, elevated TSH levels were found in 59 patients– 9.0% (95%CI: 6.4–11.6), similarly often among women– 8.5% (95%CI: 5.7–11.3) and men– 11.6% (95%: 4.6–18.5), (p = 0.37).

### Hyperthyroidism

Clinically significant hyperthyroidism was found in 120 participants (83 women and 37 men). Its prevalence was estimated at 1.6% (95%CI: 1.3–1.9) in the studied population, 2.1% (95%CI: 1.6–2.6) in women and 0.9% (95%CI: 0.5–1.3) in men (p = 0.003). The disorder was untreated in 41 (34.2%) cases with TSH < 0.1 mIU/L, including 29 women (34.9%) and 12 men (32.4%), (p = 0.79).

Hyperthyroidism was treated with antithyroid drugs in 79 subjects, but 10 of the– 12.7% had decreased TSH levels, similarly often women and men (p = 0.11).

### Untreated thyroid dysfunction

There were 201 participants (129 women and 72 men) with untreated thyroid dysfunction: 160 with hypothyroidism (21.9% of all with hypothyroidism) and 41 with hyperthyroidism (34.2% of all with hyperthyroidism). Therefore, the prevalence of untreated dysfunctions was estimated at 3.2% (95%CI: 2.5–3.8) in the whole Polish population aged 60 yrs. or above, 4.0% (95%CI: 3.0–5.1) in women and 1.9% (95%CI: 1.3–2.5) in men (p = 0.001).

**Fig 4:**
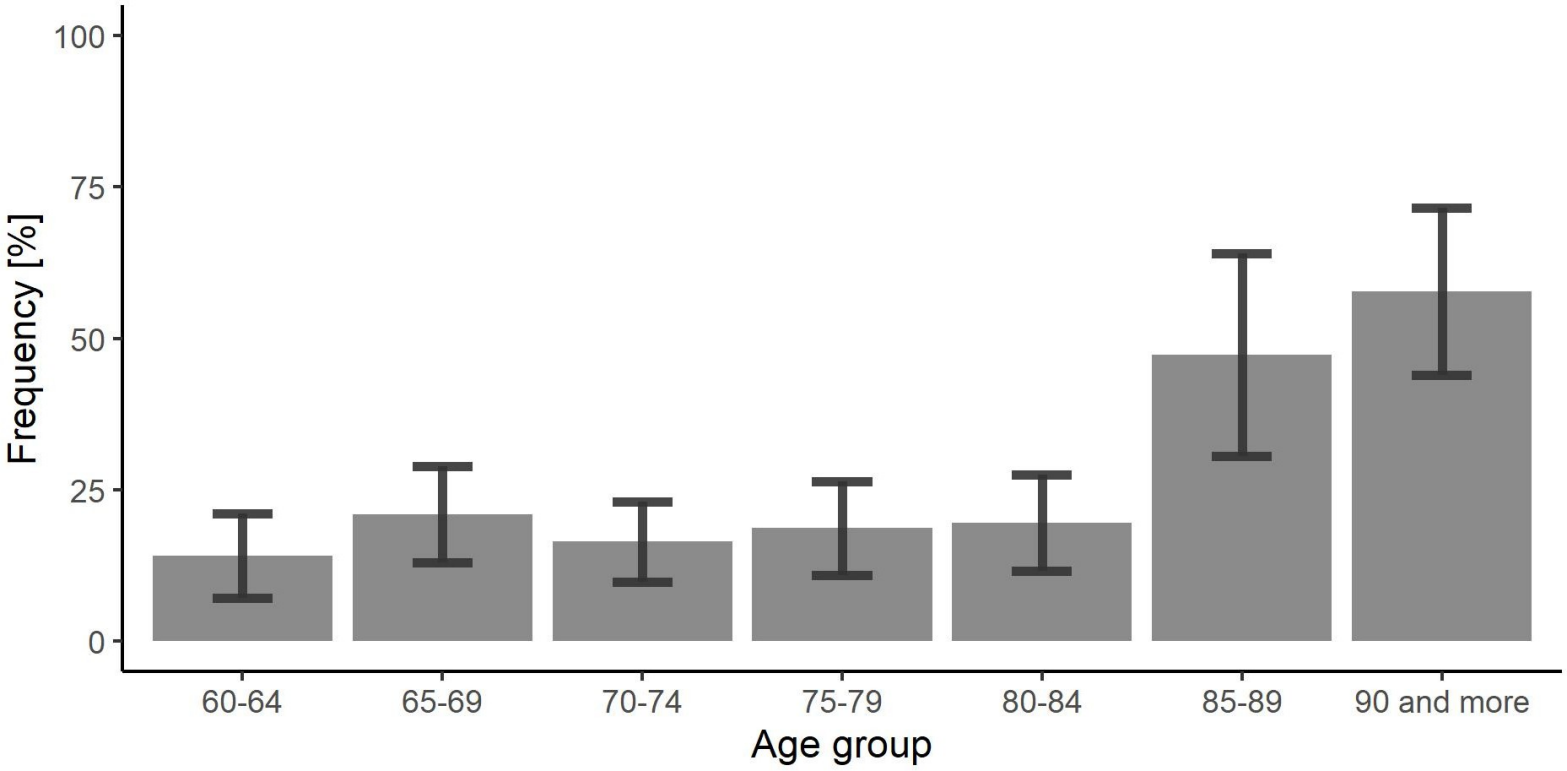
The frequency of untreated thyroid dysfunction in age cohorts.

There was a marked increase in the frequency of untreated thyroid dysfunction in the two oldest analyzed cohorts: 50.6% (95%CI: 37.5–63.7) in ≥85 yrs. old vs 17.4% (95%CI: 14.2–20.7) in 60–84 yrs. old cohorts, p < 0.001 (Fig 4).

Participants with untreated thyroid disorders were significantly older and more frequently dependent on activities of daily living (Table 1). There was a higher percentage of men among untreated ones.

**Table 1:** Comparison of subjects with treated and untreated thyroid dysfunctions.

	Treated thyroid dysfunction N = 648	Untreated thyroid dysfunction N = 201	P value
**Age [yrs]** Mean (SD); Median (IQR)	73.9 (8.6); 73 (67, 80)	79.1 (10.2); 79 (71, 87)	<0.001
**Age > 75 yrs** [N; %]	260 (40.1)	119 (59.2)	<0.001
**Women** [N; %]	496 (76.5)	129 (64.2)	<0.001
**Place of residence** [N; %]			
Village	164 (25.3)	68 (33.8)	0.05
Town < 50,000 citizens	183 (28.2)	57 (28.4)	
City 50,000–200,000 citizens	145 (22.4)	42 (20.9)	
City> 200,000 citizens	156 (24.1)	34 (16.9)	
**Marital status** [N; %]			
Never married	19 (3.0)	4 (2.0)	0.21
Married	339 (53.6)	100 (50.0)	
Widowed	245 (38.7)	91 (45.5)	
Divorced or separated	30 (4.7)	5 (2.5)	
**Living status** [N; %]			
Alone	175 (28.7)	45 (23.6)	0.10
With spouse	232 (38.1)	67 (35.1)	
Living with others	202 (33.2)	79 (41.4)	
**Education level** [N; %]			
Primary or lower	163 (25.5)	80 (39.8)	<0.001
Vocational	137 (21.4)	41 (20.4)	
Secondary	239 (37.4)	59 (29.4)	
Higher	100 (15.6)	21 (10.4)	
**Years of education** [years] Mean (SD); Median (IQR)	11.3 (3.8) 11 (8,13)	9.9 (4.3) 10 (7,12)	<0.001
**Type o work (current or in the past)** [N; %]			
‘Blue collar’ or farmer	288 (48.7)	107 (61.1)	<0.001
‘White collar’	247 (41.8)	51 (29.1)	
Other	56 (9.5)	17 (9.7)	
**Years of work** Median (IQR)	33.8 (11.0) 35 (28, 40)	35.0 (12.1) 35 (29, 42)	0.24
**Professionally active** [N; %]	41 (6.4)	5 (2.5)	0.033
**Using the Internet** [N; %]	197 (31.5)	36 (18.9)	<0.001
**Frequency of visits to the general practitioner** [N; %]			
More than once a year	601 (96.9)	165 (88.7)	<0.001
Once a year or less	19 (3.1)	21 (11.3)	
**Utilization of visits to specialists during the last 5-year period** [N; %]			
Yes	523 (82.9)	143 (72.6)	0.001
No	108 (17.1)	54 (27.4)	
**Hospitalization during the last 5-year period** [N; %]			
Yes	332 (52.6)	93 (47.0)	0.17
No	299 (47.4)	105 (53.0)	
**Income** [N; %]			
Low income	589 (93.6)	180 (90.9)	0.42
Medium income	27 (4.3)	12 (6.1)	
High income	13 (2.1)	6 (3.0)	
**iADL** [N; %]			
8–18 pts–dependent	82 (12.7)	62 (30.8)	<0.001
19–23 pts–partially dependent	113 (17.4)	31 (15.4)	
24 pts–independent	453 (69.9)	108 (53.7)	
**MMSE** (corrected) [N; %]			
27–30 pts–non-impaired cognition	407 (63.2)	112 (56.3)	0.07
24–26 pts–mild cognitive impairment (MCI)	119 (18.5)	36 (18.1)	
< 24 pts–dementia	118 (18.3)	51 (25.6)	
**GDS** [N; %]			
6–15 pts–depression	164 (28.0)	41 (25.6)	0.55
0–5 pts–no depression	421 (72.0)	119 (74.4)	

SD: Standard deviation, IQR: Interquartile range.

IADL–and Instrumental Activities of Daily Living (Lawton and Brody, 1969).

MMSE—Mini-Mental State Examination corrected by age and education according to Mungas (Mungas et al., 1996) [23].

Untreated were more frequently living in villages, had primary or lower education corresponding to lower years of education, and a higher percentage of ‘blue collars’ and farmers. Of note untreated less frequently were utilizing medical services for outpatients, both primary and specialist.

Multiple regression analysis taking into account age, sex, iADL, place of residence, education level, type of work (in the past), professional activity, cognitive function, utilization of visits to the general practitioner and specialists, and using the Internet, revealed that older age (> 75 yrs.), male sex, low education level (primary or lower) and low utilization of medical services (once a year or less) were independent risk factors for thyroid disorders being untreated (Table 2).

**Table 2:** Factors explaining the occurrence of untreated thyroid dysfunctions. There were 805 participants in the final multivariable model.

		Univariate model	Multivariable model
**Age**	> 75 yrs	2.17 (1.57–2.99)***	1.75 (1.22–2.50)**
	60–75 yrs	Reference	Reference
**Sex**	Men	1.82 (1.30–2.56)***	2.04 (1.41–2.94)***
	Women	Reference	Reference
**Place of residence**	Village	1.51 (1.07–2.12)*	-
	City	Reference	-
**Educational level**	Primary or lower	1.92 (1.39–2.70)***	1.59 (1.10–2.33)*
	other	Reference	Reference
**Type o work (current or in the past)**	‘Blue collar’ or farmer	1.80 (1.24–2.62)**	-
	Other	1.47 (0.79–2.74)	-
	‘White collar’	Reference	-
**Professionally active**	Yes	0.37 (0.15–0.96)*	0.37 (0.13–1.10)^
	No	Reference	Reference
**Using the Internet**	Yes	0.49 (0.33–0.73)***	-
	No	Reference	-
**Frequency of visits to the general practitioner**	Once a year or less	4.03 (2.11–7.67)***	4.20 (2.16–8.18)***
	More than once a year	Reference	Reference
**Utilization of visits to specialists during last 5-year period**	Yes	0.55 (0.38–0.80)**	-
	No	Reference	-
**iADL**	Dependent (8–23 pts)	2.00 (1.45–2.77)***	-
	Independent (24 pts)	Reference	-
**MMSE** (corrected)	Dementia	1.57 (1.06–2.32)	-
	Mild cognitive impairment (MCI)	1.10 (0.72–1.69)	-
	Non-impaired cognition	Reference	-

Statistical significance: ^ p < 0.1

* p<0.05

** p<0.01

*** p<0.001.

## Discussion

The results of this study allowed us to estimate the prevalence of thyroid dysfunction in the Polish senior population at 15.5%. In this age group, thyroid dysfunction is about 3-fold greater among women (21.5%), than in men (7.2%), including hypothyroidism in 13.9% (19.4% in women and 6.3% in men) and hyperthyroidism in 1.6% (2.1% in women and 0.9% in men). As expected, these figures are higher than those for the whole European population [10], most possibly due to a different age range. Previous studies reported overt and subclinical hypothyroidism in up to 5.7% and 12.5%, respectively, of older adults in various geographical areas [24–26]. Noteworthy, recent North American data showed a 24.9% prevalence of total thyroid dysfunction in people aged 65 and over [12].

The distribution of TSH values showed that the 2.5–97.5% range in the studied population, 0.28–5.38 mIU/L, is wider than the reference range provided by the test manufacturer. Established here the lower value of the low limit of the range may represent the result of common salt fortification with iodine; however, the clinical meaning of the relatively small difference seems to be important mostly from an epidemiological point of view but much less for the management of older patients in daily clinical practice.

In our study, 21.9% of subjects with hypothyroidism (18.4% of women and 31.9% of men with this pathology) and 34.2% with hyperthyroidism (24.2% of women and 32.4% of men respectively) were untreated, which corresponds to 3.2% of all Polish seniors– 4.0% of women and 1.9% of men aged 60 yrs. or above. Our data show a lower prevalence of untreated thyroid dysfunction than 6.7% [10] (with 4.9% of hypothyroidism and 1.7% of hyperthyroidism) reported for the general European population. This discrepancy may result from the increasingly frequent use of medical services by older adults, commonly suffering from chronic illnesses. Notwithstanding, the prevalence of unrecognized thyroid disease in the Australian population aged 49 years or older was 3.6% (including 3.0% of those with hypothyroidism) [27], whereas in The Colorado Thyroid Disease Prevalence Study 9.9% of the population has a functional abnormality of the thyroid gland that has been unrecognized [28]. In the latter study, however, no separate analysis for older adults was performed.

The previous study performed in the Polish senior population [11] showed a lower prevalence of thyroid dysfunction: hypothyroidism was present in 8%, and hyperthyroidism in 3% of study subjects. Among them, overt hypothyroidism and hyperthyroidism were present in 11% and 20%, respectively.

The present study shows the spectrum of factors associated with the probability of occurrence of untreated thyroid dysfunction. The strongest factor explaining the lack of treatment was the low utilization of primary health care. This proves that family doctors can effectively diagnose thyroid dysfunction and initiate treatment or referral to an endocrinologist. It was shown that a lack of insurance affecting the utilization of outpatient clinic health care may also influence the diagnosis and treatment of thyroid disorders [29]. However, even in the group with untreated thyroid dysfunction, almost 90% of subjects visited general practitioners more than once a year, which raises the need to improve physicians’ awareness of testing thyroid function in older adults.

The second strongest predictor for untreated thyroid dysfunction was male sex. Typical symptoms of thyroid dysfunction are characteristic for young patients and their occurrence predicts more accurate thyroid dysfunction in men, but clinical symptoms are less obvious and illusory in older aged patients [30]. In older subjects, the clinical manifestation of hypothyroidism might be similar to signs and symptoms associated with natural aging. The symptoms may be limited to weight gain, fatigue, muscle cramps, constipation, and skin changes [2]. The multimorbidity and adverse drug reactions can also mask or mimic the signs and symptoms of hypothyroidism. A higher percentage of undiagnosed hypothyroidism in men may suggest poor awareness of thyroid disease in men, strongly affected by up to eight to nine times lower prevalence of hypothyroidism than in women [31].

Difficulties also occur in the diagnosis of hyperthyroidism, as in the older population the most common symptom of thyroid hormone excess is tachycardia or atrial fibrillation, or exacerbation of cardiovascular disease [32]. Other symptoms of this pathology are depression, apathy, anorexia, weight loss, and fatigue. In our study, the frequency of untreated hyperthyroidism may influence the general health of the patients since untreated thyroid hormone excess may lead to the development of cardiac complications and the loss of bone mass [33] and is, therefore, more dangerous than untreated hypothyroidism, was 34.2%. Our data shows, that awareness of atypical symptoms of thyroid dysfunction and their clinical presentation in older patients seem to be insufficient for family doctors [34]. Moreover, men utilize less often visits to their primary care physician. The awareness may be effectively increased by educational programs for patients [35].

The next factor associated with undiagnosed thyroid dysfunction is the age of 75 yrs. and above that is also strongly associated with dependence on activities of daily living which, in turn, can limit access to medical services. Moreover, multimorbidity and atypical clinical manifestation of thyroid disease are also associated with age [2]. Of note, dementia was less important than the utilization of medical services.

Finally, low educational status increased the risk of undiagnosed thyroid dysfunction and improper treatment [29]. In our study group, poor education was more important than economic status. However, this may be different in other societies in which low economic status corresponds to poor insurance status and, therefore, limits the accessibility to health care systems and laboratory tests.

The results also showed that 9.0% of individuals treated for hypothyroidism and 11.6% treated for hyperthyroidism are not properly managed. The previous studies also showed that almost one-third of the patients with diagnosed hypothyroidism did not have proper hormonal substitution [26,36]. The clinical consequences of undiagnosed, untreated, or undertreated hypothyroidism seem to be especially important in patients with co-morbidities like diabetes mellitus [37] and cardiovascular disease [5]. In addition, hypothyroidism is associated with decreased quality of life [38] and increased mortality [15]. Untreated hyperthyroidism may lead to atrial fibrillation, the development of cardiovascular disease (including cardiomyopathy and heart failure), bone loss, and thyroid storm [39]. Notwithstanding, clinical consequences of subclinical hypothyroidism are controversial, especially in the oldest. Even though the recent data negate the benefits of levothyroxine supplementation [40].

Among the limitation of our study, the lack of the testing of the agreement between interviewers due to their large number has to be mentioned.

## Conclusions

Physicians should be aware of possible thyroid dysfunction in their older patients of both sexes. The unanswered question remains on the purposefulness of screening thyroid function in the whole senior population. The results of our study are important in the view of a high frequency of thyroid dysfunction in the elderly population. Moreover, the percentage of untreated thyroid dysfunction is quite high suggesting that a huge number of patients still are left without treatment which may have a detrimental effect on their health.

## Supporting information

S1 File (DOC)
