# Supplementary material for: Prevalence and risk factors of untreated thyroid dysfunctions in the older Caucasian adults: Results of PolSenior 2 survey
Source: PLoS One. 2022 Aug 22;17(8):e0272045. doi: 10.1371/journal.pone.0272045 (PMC9394816; doi:10.1371/journal.pone.0272045)
Supplement: S1 File — (DOC) [file pone.0272045.s001.doc]

The response to the Journal Requirements:

1. The list of authors and affiliations is correct.
2. Funding information is as follows: “This paper was implemented under contract No. 6/5 / 4.2 / NPZ / 2017/1203/1257 for the implementation of the task in the field of public health of the Operational Objective No. 5 points 4.2. of the National Health Program for years 2016-2020, entitled "Health Status and Its Socio-economic Covariates of the Older Population in Poland - the Nationwide PolSenior2 Survey" (PolSenior2).
   The funders had no role in study design, data collection and analysis, decision to publish, or reparation of the manuscript.”
3. Data Availability Statement - The minimal data set is available at <https://polsenior2.mug.edu.pl/68966.html>
4. The proper names of the authors are: Monika Puzianowska-Kuźnicka and Adrian Stefański. The names are correct in the manuscript, please correct them in the submission form.
5. Downable sample files show correct affiliations and byline formation.
6. The Author Formatting Checklist helped us to confirm the requirements for References, Tables, and Figures.
7. The PACE tool assessed the Figures which meet the technical requirements.

JOURNAL REQUIREMENTS:

1. Please ensure that the author list and affiliations are correct on the title page of your manuscript, and that your author contributions, competing interests, and financial disclosure are correct as listed below. All of these sections will be indexed in PubMed and published by PLOS ONE as you have written them. Please email plosone@plos.org if any changes to this content need to be made.

Piotr Kocełak:
Conceptualization
Formal analysis
Methodology
Writing – original draft

Małgorzata Mossakowska:
Writing – original draft
Writing – review & editing

Monika Puzianowska-Kuznicka:
Writing – review & editing

Krzysztof Sworczak:
Supervision

Adam Wyszomirski:
Data curation
Software

Gabriela Handzlik:
Writing – review & editing

Adrian Stefanski:
Project administration
Software

Tomasz Zdrojewski:
Funding acquisition
Methodology
Project administration
Resources

Jerzy Chudek:
Conceptualization
Data curation
Methodology
Supervision
Validation
Writing – original draft
Writing – review & editing

Please see here for the full list and definition of contributor roles: http://journals.plos.org/plosone/s/authorship#loc-author-contributions

Please ensure that the Competing Interests and Financial Disclosure statements listed below are suitable for publication. These sections will be indexed in PubMed and published by PLOS ONE as you have written them. Please email plosone@plos.org if any changes to these statements need to be made.

Competing Interests:
The authors have declared that no competing interests exist.

Financial Disclosure:
This paper was implemented under contract No. 6/5 / 4.2 / NPZ / 2017/1203/1257 for the implementation of the task in the field of public health of the Operational Objective No. 5 points 4.2. of the National Health Program for years 2016-2020, entitled "Health Status and Its Socio-economic Covariates of the Older Population in Poland - the Nationwide PolSenior2 Survey" (PolSenior2).
The funders had no role in study design, data collection and analysis, decision to publish, or reparation of the manuscript.

2. We note that the funders listed in your submission's Funding Information do not match the information given in your Financial Disclosure statement. Your current Funding Information lists the following funders:

ministerstwo nauki i szkolnictwa wyższego, 6/5 / 4.2 / NPZ / 2017/1203/1257, Professor Tomasz Zdrojewski

Your current Financial Disclosure statement is as follows:

This paper was implemented under contract No. 6/5 / 4.2 / NPZ / 2017/1203/1257 for the implementation of the task in the field of public health of the Operational Objective No. 5 points 4.2. of the National Health Program for years 2016-2020, entitled "Health Status and Its Socio-economic Covariates of the Older Population in Poland - the Nationwide PolSenior2 Survey" (PolSenior2).
The funders had no role in study design, data collection and analysis, decision to publish, or reparation of the manuscript.

By return email, please indicate the full and correct list of funders for your study. We will update your submission on your behalf according to the instructions you provide.

3. We note your current Data Availability Statement: "The data can be obtained from the corresponding author"

Please note that in the interest of long-term data availability, PLOS data policy states that it is not acceptable for an author to be the sole named individual responsible for ensuring data access. (https://journals.plos.org/plosone/s/data-availability#loc-acceptable-data-access-restrictions)

Please also note that PLOS only allows for data to be available upon request if there are acceptable ethical, legal, or third party restrictions. If there are such restrictions, please provide the following information (https://journals.plos.org/plosone/s/data-availability#loc-acceptable-data-access-restrictions):
1. A complete description of the dataset
2. The nature of the restrictions upon the data (ethical, legal, or owned by a third party) and the reasoning behind them
3. The full name of the body imposing the restrictions upon your dataset (ethics committee, institution, data access committee, etc)
4. If the data are owned by a third party, confirmation of whether the authors received any special privileges in accessing the data that other researchers would not have
5. Direct, non-author contact information (preferably email) for the body imposing the restrictions upon the data, to which data access requests can be sent

This information will be helpful in updating your Data Availability Statement.

4. Author Monika Puzianowska-Kuźnicka is listed as Monika Puzianowska-Kuznicka in the online submission form; we are unsure which version is correct. Author Adrian Stefański is listed as Adrian Stefanski in the online submission form; we are unsure which version is correct. If the names are incorrect in the manuscript, please correct this. If the names are incorrect in the submission form, please let us know so we can correct the author’s profile for you. Please note that the use of middle initials/names should be consistent among the manuscript’s author list, the author list in the submission form, and the Author Contributions initials.

5. Please refer to our downloadable sample files to make sure that your affiliations and byline are formatted correctly: http://journals.plos.org/plosone/s/file?id=ba62/PLOSOne_formatting_sample_title_authors_affiliations.pdf

6. To prevent production delays, we recommend using the Author Formatting Checklist to confirm that your paper meets PLOS ONE's typesetting requirements for References, Tables, and Figures: http://journals.plos.org/plosone/s/file?id=c819/plos-one-author-formatting-checklist.docx.

This checklist is a reference tool for you; please do not upload the completed Author Formatting Checklist with your submission files.

7. To ensure your figures meet our technical requirements, please run each figure included in your submission files through the PACE tool: https://pacev2.apexcovantage.com/. PACE will assess whether your figures meet our technical requirements and will fix the figure(s) or identify any problem(s) that cannot be automatically fixed. It can also convert figures to TIFF format, resize, and rename figures to meet our naming conventions.

To use PACE, first register as a user. Follow the instructions on the site for assessing and converting your figure files. If you experience any difficulty using this tool or have questions about any of the figures and/or images in your paper, please inform the journal office in your response letter.

CONFIDENTIAL: This email and any attachments are confidential and for the sole use of the individual(s) to whom they are addressed. If you have received this message in error please delete the message and notify plosone@plos.org.
